# The Evolution of Annual Immunization Recommendations Against Influenza in Italy: The Path to Precision Vaccination

**DOI:** 10.3390/vaccines13040356

**Published:** 2025-03-27

**Authors:** Sara Boccalini, Chiara de Waure, Linda Martorella, Paolo Orlando, Paolo Bonanni, Angela Bechini

**Affiliations:** 1 Department of Health Sciences, University of Florence, 50134 Florence, Italy; linda.martorella@unifi.it (L.M.); paolo.orlando@unifi.it (P.O.); paolo.bonanni@unifi.it (P.B.); angela.bechini@unifi.it (A.B.); 2 Department of Medicine and Surgery, University of Perugia, 06132 Perugia, Italy; chiara.dewaure@unipg.it

**Keywords:** appropriateness, vaccine, risk group, elderly, children, pregnancy, adjuvanted vaccine, high dose vaccine, cell-based vaccine, influenza live-attenuated vaccine

## Abstract

Influenza vaccination is the health intervention that best guarantees protection against seasonal influenza. Every year, the Ministry of Health issues a document (namely a circular) containing recommendations for the use of available vaccines to prevent and control influenza. A review of the Ministerial Circulars released by the Italian Ministry of Health was conducted with the aim of assessing the evolution over time of the vaccination target recipients, the vaccines that can be used in the following influenza season, and the indications of use for age and appropriateness. Changes have emerged regarding these issues. In fact, over time, the vaccination offer has been extended to children between 6 months and 6 years of age, to adults aged 60–64 years, and to women in any trimester of pregnancy and postpartum. In parallel, from the 2018–2019 season, following the availability of new vaccines, the concept of vaccine appropriateness was introduced to the recommendations, i.e., the choice of the most appropriate vaccine for each subject to be vaccinated. The last circulars introduced the indication of the type of vaccine that can be administered for each target category and the recommendation to use adjuvanted and high-dose vaccines for adults aged ≥ 65 years. The annual recommendations provided by the Italian Ministry of Health, resultantly, are increasingly precise, emphasizing the importance of the concept of appropriateness and outlining the path to precision vaccination. To achieve maximum value in terms of clinical efficacy and community benefits, this concept should continue to be considered as a basis for the development of future recommendations, as it addresses a critical public health issue related to vaccination.

## 1. Introduction

Influenza is a viral infectious disease of the respiratory tract that is preventable through vaccination. Influenza exhibits a seasonal epidemiological pattern with epidemic characteristics. Indeed, in temperate climates of the northern hemisphere, seasonal epidemics mainly occur during winter, whereas in tropical regions, influenza seasonal epidemics can occur throughout the year, leading to more irregular outbreaks. Seasonal influenza represents a global challenge for public health due to its significant impacts on both individuals and society. It is estimated that globally, every year, there are approximately one billion cases of influenza, with 3–5 million severe symptoms and 290,000–650,000 deaths, as reported in the *Global Influenza Strategy* 2019–2030 [1,2].

Influenza is caused by various RNA viruses belonging to the Orthomyxovirus family. Influenza A and B viruses are the main cause of seasonal epidemics and classical influenza symptomatology [1,2,3]. Influenza viruses are characterized by a pronounced tendency to mutate genetically, resulting in the presentation of antigenic variants of the two virus A glycoproteins hemagglutinin (HA) and neuraminidase (NA), allowing the virus to evade the host’s immune response developed following previous infections. For this reason, most of the population is immunologically susceptible to new influenza variants, and consequently, these can spread easily [4]. Influenza B virus is not classified into subtypes according to antigenic variants but can be divided into the two lineages Yamagata and Victoria. Generally, B virus tends to cause less severe forms of influenza compared to type A influenza virus [1].

Influenza can have an asymptomatic course (up to 75% of infections during the influenza season), be symptomatic without complications, or lead to a severe clinical picture [5].

Although influenza affects individuals of all age groups, some categories are at higher risk of infection [6], such as children and adolescents [7], while others are more likely to develop complications (very young children, individuals with chronic diseases, and older subjects). The population at greatest risk of developing influenza-related complications, as well as being hospitalized or experiencing death, is the elderly (≥65 years old), especially those with chronic diseases. However, although the greatest *burden* of disease affects the older population, it is important to note that influenza can also lead to complications and hospitalizations in children and adults [3,8]. As a matter of fact, in general, the incidence of influenza-associated complications is lower in the pediatric age group than in the older population, but it is not negligible. The most important risk factors for the development of influenza-associated complications in young individuals are age under 5 years and the presence of chronic diseases. Ninety-nine percent of deaths occur in developing countries [9].

Vaccination is the most effective preventive intervention against influenza. International and national health authorities actively engage in the development of effective prevention strategies, and the recommendation of influenza vaccination plays a central role in these efforts. Currently, various types and formulations of influenza vaccines are available, characterized by a specific profile of efficacy and safety. These different types of vaccines have different indications for use based on age [10,11].

In Italy, each year, the Ministry of Health prepares a Ministerial Circular for the prevention and control of seasonal influenza. This document, in addition to providing information on epidemiological and virological surveillance during the just-ended season, provides both indications on the viral composition of vaccines that will be available for the next season and indications for use based on age (as provided by the Summary of Product Characteristics—SPC) and identifies the categories of people for whom influenza vaccination is recommended. The recommendation of seasonal influenza vaccination aims to reduce the individual risk of disease, hospitalization, and death, reduce the risk of virus transmission to persons at high risk of complications and/or hospitalization, and reduce the social costs related to morbidity and mortality [12]. Therefore, in Italy, as well as internationally, the main recipients of seasonal influenza vaccination should be older subjects (aged 65 years or older) but also people of all ages with underlying pathologies that increase the risk of developing complications as a result of influenza and health professionals. Nonetheless, because of the growing availability of different types of vaccines, it is evident that the maximum preventive benefit can be obtained with *tailor-made* vaccination recommendations specific to each population group to offer the best result in terms of immunogenicity, efficacy, and safety.

This study aims to examine and summarize the evolution of national recommendations for influenza prevention in Italy with the final objective of providing a thorough and updated overview that could be considered the foundation for the development of future recommendations.

## 2. Materials and Methods

The Italian Ministerial Circulars for the prevention and control of seasonal influenza from the 2013–2014 season to the 2024–2025 season were searched on the Ministry of Health website [13] and collected. Each circular was analyzed in all its sections, focusing on the target categories for which seasonal influenza vaccination is recommended and offered actively and free of charge, in accordance with the objectives of national health planning and with the pursuit and specific objectives of the influenza immunization program, and on the available vaccines authorized in Italy for the influenza season on the basis of the indications by age, as reported by the SPC. In addition, any indications of the preferential and appropriate use of available vaccines based on risk category were researched. Once the key information for each epidemic season had been collected, these three aspects were collected in a database and evaluated over time, highlighting similarities and differences between the recommendations for one epidemic season and the next with the use of summary tables.

## 3. Results

### 3.1. Target Population for Vaccination

In the 2013–2014 season, the Italian Ministry of Health’s annual Ministerial Circular for influenza prevention [14] recommended influenza vaccination, actively and free of charge, to specific target categories. These groups remained largely unchanged in the Ministerial Circulars for the subsequent seasons, with some integrations [12,15,16,17,18,19,20,21,22,23,24].

These Ministerial Circulars prioritized influenza vaccination for individuals deemed to be at a high risk of serious influenza-related outcomes. This group included adults aged 65 years and older; individuals aged 6 months to 65 years with conditions such as chronic respiratory, cardiovascular, metabolic, renal, hematologic, oncologic, or immunodeficiency disorders; children and adolescents on long-term treatment with acetylsalicylic acid; residents of extended-care facilities; family and close contacts of high-risk individuals; and pregnant women in their second and third trimesters at the beginning of the influenza season.

Immunization against influenza was further recommended for individuals engaged in public services of critical collective interest and specific occupational categories, primarily medical and healthcare staff who, through their activities, are able to transmit influenza to individuals considered to be at high risk. Other critical occupational groups are police and fire services and other personnel whose work-related activities would be enhanced by vaccination. Furthermore, vaccination was provided for those whose professional duties involved contact with animals susceptible to non-human influenza viruses, including agricultural workers, livestock transporters, and veterinary professionals.

Starting from the 2018–2019 season, some integrations and modifications in the target population for vaccination were introduced. In the Ministerial Circular for the 2018–2019 season [19], blood donors were indeed added to the target categories for whom seasonal vaccination is recommended and free of charge. Another substantial modification concerns pregnant women: the recommendation for vaccination was extended to all trimesters of pregnancy starting in the Ministerial Circular for the 2019–2020 season because seasonal influenza increases the risk of hospitalization, abortion, prematurity, and low birth weight, and subsequently, the postpartum period was also included in the target group [20,25].

Regarding the pediatric population, influenza vaccination has been also recommended in Italy for healthy children in the age range of 6 months to 6 years only in recent years. In fact, until the Ministerial Circular for the 2019–2020 season, influenza vaccination was recommended only for children at risk of influenza complications due to pre-existing conditions. In the pandemic context, starting from the 2020–2021 season, initially with a general recommendation (referring to the scientific evidence of the direct and indirect impact of pediatric influenza immunization in the world) and subsequently in the following seasons with specific stronger and clearer recommendations, the Ministry of Health identified the healthy pediatric population as another of the groups to whom the offer of influenza vaccination should be extended. The aim of this recommendation was also to reduce influenza virus circulation among adults and older subjects. Also, in the Ministerial Circular for the 2020–2021 season, due to the COVID-19 emergency, the possibility of offering free of charge influenza vaccination to the 60–64 years age group, regardless of the presence of risk situations, was given to facilitate differential diagnosis in age groups at greater risk of severe illness [21]. The 2024–2025 Ministerial Circular revised the classification, placing individuals aged 60–64 years and healthy children aged 6 months to 6 years within the high-risk group for influenza complications and hospitalizations, rather than in other recommended vaccination categories [12].

Table 1 details the groups now recommended for free influenza vaccination, in accordance with the 2024–2025 Ministerial Circular [12].

### 3.2. Vaccines Available for Use

Each year, the Ministerial Circular reports a list of influenza vaccines available, authorized by the Italian Medicines Agency (AIFA), and the indications for their use by age group based on the SPC. The types and formulations of vaccines have changed over the years, as indicated in Table 2 [12,14,15,16,17,18,19,20,21,22,23,24].

In the Ministerial Circulars for epidemic seasons from 2013–2014 to 2017–2018, the available influenza vaccines were mainly inactivated split or subunit (trivalent or quadrivalent) vaccines, an adjuvanted inactivated trivalent vaccine with MF59, and an intradermal inactivated trivalent vaccine (split type) (IDTIIV) [14,15,16,17,18]. Starting from the Ministerial Circular for the 2014–2015 season, the virosomal adjuvanted inactivated trivalent vaccine (VATIIV) was no longer available [15]. In the 2013–2014 and 2014–2015 seasons, the circulars reported that an inactivated vaccine produced on cell cultures and an intranasal live attenuated vaccine were also authorized but specific indications of use were not included in the documents [14,15].

Furthermore, in the Ministerial Circular for the 2015–2016 season, the inactivated trivalent vaccine produced on cell cultures was officially indicated among usable vaccines but was no longer available from the following seasons, as reported in the circulars for the 2016–2017 and 2017–2018 seasons [16,17,18].

The intradermal inactivated trivalent vaccine (split type) was no longer reported from the Ministerial Circular for the 2018–2019 season [19]. Additionally, the quadrivalent live attenuated vaccine administered intranasally (LAIV) was reported among those authorized in Italy in the Ministerial Circular for the 2018–2019 season [19], even though the specific indications for use by age group were available only later, in the Ministerial Circular for the 2021–2022 season [22].

The quadrivalent vaccine produced on cell cultures (QIVcc) was indicated among those available from the Ministerial Circular for the 2019–2020 season onwards, the high-dose quadrivalent vaccine (QIVhd) was indicated starting from the 2020–2021 season, and, lastly, the recombinant quadrivalent vaccine (rQIV) was indicated in the Ministerial Circular for the 2021–2022 season onwards [20,21,22]. This last vaccine, even if it is authorized and recognized as a vaccine usable in the influenza vaccination campaign, de facto is not available in Italy.

All influenza vaccines were trivalent in the Ministerial Circular for the 2013–2014 season; later, inactive quadrivalent vaccines started to become available (especially split ones). All usable vaccines were quadrivalent from the Ministerial Circular for the 2021–2022 season [22], but in the Ministerial Circular for the 2024–2025 season, LAIV is trivalent, according to WHO indications [12]. In fact, since no cases attributable to the B/Yamagata influenza virus have been reported among circulating viruses globally since March 2020, this lineage is no longer considered a public health risk, and vaccination against this virus is not regarded as necessary [26]. Therefore, the Emergency Task Force (ETF), with the endorsement of the Committee for Medicinal Products for Human Use (CHMP) of the European Medicines Agency (EMA), has recently recommended excluding the B/Yamagata-related vaccine component from influenza vaccine compositions in the near future. The transition from quadrivalent to trivalent vaccines should be implemented by the 2025–2026 season, except for the trivalent live attenuated vaccine, which should be available as early as the 2024–2025 season [12].

Therefore, currently, the following influenza vaccines are authorized and potentially usable in Italy for the Ministerial Circular for the 2024–2025 season: standard-dose inactivated (split or subunit) quadrivalent vaccines produced on eggs (QIV), a live intranasal attenuated trivalent vaccine (LAIV), a cell-based quadrivalent vaccine (QIVcc), a recombinant quadrivalent vaccine (rQIV) (de facto not available in Italy), a high-dose quadrivalent egg-based vaccine (QIVhd), and an egg-based adjuvanted quadrivalent vaccine (aQIV) [12].

Table 3 shows the types of influenza vaccines that could be administered in the different age groups, according to the SPC, as reported in the annual circulars for the prevention and control of influenza in the different epidemic seasons from 2013–2014 to 2024–2025 [12,14,15,16,17,18,19,20,21,22,23,24].

Inactivated vaccines are administrable to younger subjects, even if LAIV and QIVcc vaccines have become available for subjects ≥2 years. Inactivated virosomal and intradermal vaccines were manageable in an adult population in the past. Nevertheless, VATIIV and IDTIIV have become unavailable in Italy over time and have been replaced by QIVcc and rQIV. The older population has always had the greater number of administrable vaccines, starting from the administration of TIV, VATIIV, IDTIIV, and aTIV, now QIV, QIVcc, rQIV, QIVhd, and aQIV may be supplied to this age group. Recently, aQIV underwent some changes in the SPC, being currently usable in subjects ≥50 years [12].

### 3.3. Indications of Use

Until the 2017–2018 influenza season, Ministerial Circulars did not include any indication as to which vaccine was preferable and appropriate to use among those available. In these documents, only the doses and administration method of each vaccine based on age were reported, according to the SPC [14,15,16,17,18]. From subsequent Ministerial Circulars (2018–2019 season and onwards), indications of preferential use among available vaccines for each age group were reported [12,19,20,21,22,23,24].

The Ministerial Circular for the 2018–2019 season recommended the use of the inactivated quadrivalent influenza vaccine (QIV) as the preferred formulation for children and adolescents. In the case of unavailability, the use of the non-adjuvanted trivalent split or subunit vaccine (TIV) was suggested. For adults with chronic health conditions and healthcare workers, both TIV and QIV could be administered, but the quadrivalent formulation was preferred. Pregnant women were indicated to receive either the trivalent or quadrivalent formulation. Adults aged ≥ 65 years could be administered non-adjuvanted and adjuvanted trivalent vaccines (TIV or aTIV) as well as QIV, but for those over 75 years, the adjuvanted TIV was recommended preferentially due to its higher efficacy in this age group compared to the other two types of influenza vaccines [19]. The same preferential use indications were essentially repeated in the Ministerial Circular for the 2019–2020 season [20].

In the Ministerial Circulars for the three seasons 2020–2021, 2021–2022, and 2022–2023, the indication for the preferential use of vaccines has been increasingly reduced and made more generic, including only indications based on age as reported in the SPC for each vaccine [21,22,23]. On the contrary, the Ministerial Circular for the 2023–2024 season contains a significant innovation (confirmed in the circular for the 2024–2025 season), namely a new table indicating the type of vaccine that can be administered to different categories eligible for vaccination, and, above all, a preference indication for use, where possible (Table 4) [12,24]. From the table, it is evident that the high-dose quadrivalent vaccines produced in eggs (QIVhd) and those adjuvanted with MF-59^®^ (aQIV) are the most appropriate for use in subjects aged ≥ 65 years.

## 4. Discussion

The aim of this study was to describe the evolution of national recommendations for influenza prevention in Italy from the 2013–2014 to the 2024–2025 season. The results of the study highlight how there have been changes regarding the target population for the influenza vaccination program and the vaccines available for use, concurrently with technological advancements in the field and in terms of indications for the preferential use of different vaccine types.

In Italy, there has been an expansion of the vaccines offered over the years, particularly targeting children aged between 6 months and 6 years and progressively extending to pregnant women in any trimester and in the postpartum period and adults 60–64 years old. At the same time, in recent years, research in the field of influenza prevention has led to the development of new vaccine platforms with different efficacy/effectiveness, specifically tailored to different age groups to maximize health and quality of life outcomes and economic savings and ensure adequate protection for the entire community. Therefore, with the increasing number of available influenza vaccines, the challenge is the best use of them to reach greater health value for the population.

Until the 2017–2018 influenza season, there were no references to the preferable use of the available vaccines. The concept of appropriateness [27,28], namely the choice of the most appropriate vaccine for the individual to be vaccinated, has only recently become part of institutional recommendations with the Ministerial Circular for the 2018–2019 influenza season [24]. Today, there is much evidence justifying the concept of appropriateness for influenza vaccines based on epidemiological, immunological, and clinical data and many Health Technology Assessment (HTA) reports on influenza vaccination in Italy [9,29,30,31,32,33,34,35].

Based on the evidence, the circular for the 2018–2019 season introduced recommendations for the use of some vaccines relative to specific target groups. In particular, the use of the quadrivalent formulation of the inactivated vaccines compared to the trivalent ones in children and adolescents and the vaccine adjuvanted with MF59 for adults aged ≥ 65 years. The first indications are related to the fact that the ratio between the percentage of influenza-causing virus types for a specific age group and the percentage representation of that same age group within the general population confirms that in the age range of 0 to 17 years, the B virus has the highest frequency, while among those over sixty-five, it is the AH3N2 subtype [36]. As a matter of fact, the main factor impacting protection against influenza B strains is previous exposure to this strain. With increasing age, the impact of B lineage mismatch (i.e., the possibility of circulating a lineage B not contained in the vaccine) on vaccine efficacy decreases. This means that children and young adults, often naive to the B virus, will derive greater benefits from the use of a quadrivalent vaccine compared to trivalent ones, while older adults will have fewer benefits, as they have been in contact with the B virus multiple times in the past [37]. On the other hand, the recommended use of vaccine adjuvanted with MF59 for adults aged ≥ 65 years is due to numerous studies that have extensively demonstrated the superior effectiveness of adjuvanted influenza vaccine compared to non-adjuvanted vaccine [38,39,40,41,42,43,44].

However, in the following years, new influenza vaccines have been introduced into the market, and consequently, the possible options for administration for various age groups have changed and increased. The quadrivalent cell-culture-based vaccine (QIVcc) has been commercialized in Italy from the 2019–2020 season onwards. This vaccine is produced through a process that reduces mutations due to virus adaptation to eggs (egg-adaptive mutations), which can result in antigenic mismatch between circulating and vaccine strains, leading to suboptimal vaccine efficacy [45,46,47,48,49].

The quadrivalent high-dose vaccine (QIVhd) has been authorized since the 2020–2021 season in Italy [21]. The high-dose vaccine has been shown to be superior to the standard-dose vaccine in terms of efficacy and effectiveness in the older population in reducing both influenza cases and clinical complications associated with the infection, regardless of the circulating strain and antigenic match [50,51,52,53,54].

The last influenza vaccine authorized in Italy in subjects aged 18 years and older, as indicated in the circular for the 2021–2022 season onwards, was the quadrivalent recombinant DNA vaccine (rQIV). It represents another vaccine developed with a technology that allows, in addition to eliminating the possibility of mutations associated with virus propagation in eggs, a faster production speed with higher overall yield and the ability to negate the potential risk of egg albumin allergies in atopic individuals [34,55,56,57].

A randomized controlled clinical trial evaluating the immunogenicity of standard-dose, high-dose, MF59-adjuvanted, and recombinant influenza vaccines in the elderly population (65–82 years) showed that enhanced (high-dose and adjuvanted) influenza vaccines increase the polyfunctional CD4+ and CD8+ T-cell response. These results, therefore, provide further immunological evidence for the preferential use of QIVhd and aQIV in the elderly population [58]. The main cited studies that demonstrate the scientific evidence behind the preferential use of QIVhs and aQIV in the elderly population are summarized in Table 5.

Therefore, QIVhd and aQIV are the two vaccines specifically recommended among the available vaccines for the older population aged ≥ 65 years in the latest Ministerial Circulars. Considering the greater efficacy against symptomatic disease and the slightly higher reactogenicity of recombinant, adjuvanted, and high-dose vaccines compared to standard inactivated vaccines in the elderly, the WHO specifically recommends their use for this particularly vulnerable group, as they can provide additional protection [59].

In the 2021–2022 influenza season, an intranasal live attenuated vaccine was added to the injectable vaccines for the pediatric population. The way of administration is a feature particularly important for promoting compliance in the younger population (2–18 years). This vaccine has been shown to provide a broader immune response compared to that stimulated by inactivated vaccines, both against homologous and heterologous influenza viruses, more durable over time, and capable of providing protection for the entire influenza season [34].

Enhancing public health offerings through a value-based approach can elevate the quality of healthcare services and ensure their long-term sustainability. Indeed, the administration of the influenza vaccine is essential to prevent complications, hospitalizations, and deaths. However, choosing the most suitable vaccine for each target group maximizes the benefits at a minimum cost [11].

Other countries are also moving towards precision vaccination. Germany and Australia have expressed preferential recommendations for the use of boosted (adjuvanted or high-dose) vaccines in older subjects, recognizing their superior effectiveness, which justifies the investment in terms of the cost of the doses against even higher savings in terms of cases and complications avoided. In Germany, the Standing Committee on Vaccination (STIKO) recommends the high-dose inactivated quadrivalent vaccine (QIVhd) for adults aged ≥60 years [60]. The National Immunisation Program (NIP) in Australia, on the other hand, recommends the administration of the quadrivalent inactivated adjuvanted vaccine (aQIV) in adults aged ≥ 65 years [61].

The National Health Service (NHS) of the United Kingdom recommends the preferential administration of the recombinant quadrivalent vaccine (rQIV) in adults aged ≥ 65 years over the adjuvanted quadrivalent inactivated vaccine (aQIV), which is also available for this age group [62]. Preliminary UKHSA evidence in the UK from the 2022 to 2023 influenza season and international evidence suggests that the potential additional benefit may be greatest in adults aged 65 years and older [63,64].

The United States of America (USA) and Canada, on the other hand, do not provide indications for the preferential use of available influenza vaccines for individuals aged ≥ 65 years. The Advisory Committee on Immunization Practices (ACIP) of the USA recommends that adults aged ≥ 65 years should preferably receive one of the following: high-dose inactivated quadrivalent vaccine, recombinant quadrivalent vaccine, or adjuvanted inactivated quadrivalent vaccine [65]. The National Advisory Committee on Immunisation (NACI) of Canada recommends one of the following vaccines for use in adults aged ≥ 65 years: the trivalent inactivated adjuvanted vaccine (aTIV), the quadrivalent inactivated standard-dose vaccine (QIV), the quadrivalent inactivated cell-culture vaccine (QIVcc), the quadrivalent inactivated high-dose vaccine (QIVhd), or the quadrivalent recombinant vaccine (rQIV) [66].

**Figure 1:**
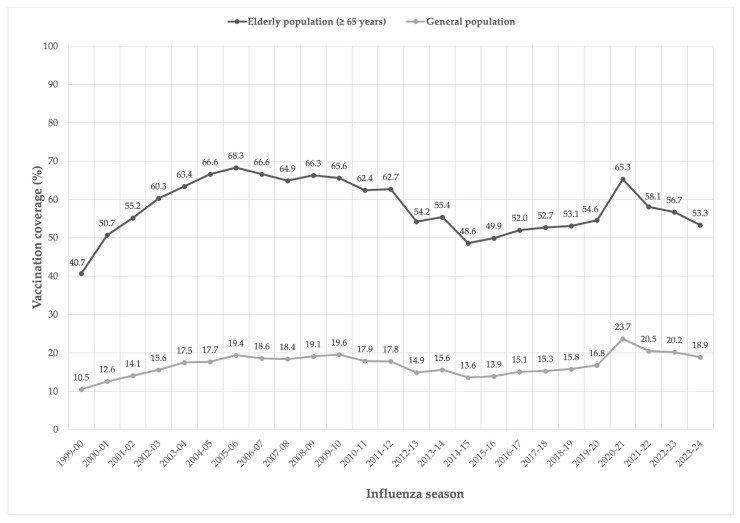
Influenza vaccination coverage rates in Italy for the period 1999–2023 [68].

However, the appropriateness of use for influenza vaccines becomes much more important in some countries each year. On the other hand, today, the challenge is to increase the number of administrations, namely vaccine coverage. In Italy, the current National Immunization Plan (NIP 2023-2025) and the annual Ministerial Circulars have set the minimum vaccination coverage goal at 75% and the optimal one at 95% for subjects over sixty-five years and high-risk groups [67]. Unfortunately, vaccination coverage rates in Italy are far from these objectives. Vaccination coverage has never exceeded 70% in the elderly population (≥65 years), while in the general population, it ranges between 15% and 20%. For the 2022–2023 season, the vaccination coverage for the general population was 20.2%, stable compared to the previous season (data as of 20 July 2023). Analyzing the trend of influenza vaccination coverage in the elderly reported by the Ministry of Health for the period 1999–2023, there is a minimum value of 41.7% for the 1999–2000 influenza season. The data show a positive trend, reaching 68.3% in the 2005–2006 season. This was followed by a decline in vaccination coverage to 48.6% recorded in the 2014–2015 season. Since the 2015–2016 season, there has been a constant increase in vaccination coverage, reaching 65.3% in the 2020–2021 season. In the 2021–2022 season, coverage in the elderly decreased compared to the previous season (58.1%) and decreased again marginally (56.7%) in the 2022–2023 season (Figure 1) [68].

Although reports on vaccination coverage trends have been published, most of these focus on epidemiological data, without investigating the reasons behind the decline in vaccination coverage over the last three influenza seasons. However, some considerations can be made. The decline in influenza vaccination coverage in Italy between 2021 and 2023 can be attributed to multiple factors, some of which are related to changes in the social and healthcare context, while others reflect more specific dynamics concerning the perception of vaccination itself. During the COVID-19 pandemic, the influenza vaccination campaign experienced a significant increase, as the population was more aware of and inclined toward vaccination. Afterwards, the perceived urgency for influenza vaccination began to decline and vaccination coverage returned to levels more similar to those observed before the pandemic. Many resources (both financial and human) were allocated to the health emergency, sidelining awareness and promotional campaigns for influenza vaccination. The reduction in public communication regarding the importance of the influenza vaccine may have contributed to lower adherence, particularly among the elderly population. Additionally, between 2021 and 2023, the perceived risk of contracting influenza was lower, primarily due to public health measures such as social distancing and mask-wearing, which reduced the circulation of many respiratory viruses.

Therefore, in the future, the great challenge will be to increase vaccination coverage in the target groups through promotional activities and, concurrently, to promote the use of the most appropriate vaccines.

The availability of official indications on the best use of vaccines can support healthcare workers in immunization activities and enhance the confidence of both physicians and patients in the vaccination process. Ensuring that physicians are up to date on the latest recommendations and the characteristics of various vaccines may contribute to more effective patient counseling, thereby increasing their willingness to get vaccinated [69,70,71]. Other potential strategies to improve vaccination adherence rates could include an active call system (letters, SMS, e-mail, or phone calls) for the vaccination offer, providing a constant offer by avoiding vaccine shortages in health centers, facilitating access to vaccination (e.g., without appointments in pharmacies), and implementing proximity strategies for vaccination in workplaces and schools [72,73].

In addition to specific training measures for general practitioners and pediatricians and the implementation of vaccination strategies (the accessibility, distribution, and availability of vaccines), targeted educational activities for the population will also be essential for achieving this goal [74,75,76].

### Limitations of the Study

A review of Ministerial Circulars may provide a useful overview of the evolution of Italian vaccination policies, but it is important to consider the intrinsic limitations of this type of study. The study cannot assess the actual implementation of the recommendations as it must be considered that not all the vaccines authorized for use are available on the commercial market. The population’s adherence to vaccination is also not an outcome that can be assessed.

The language and form in which these documents were drafted varied slightly over time, and the information was sometimes not entirely comprehensive, which made comparison more difficult.

## 5. Conclusions

A comparison of influenza vaccination recommendations for each epidemic season revealed changes over time in the three key aspects analyzed: vaccination recipients, the availability of administrable vaccines and indications for use by age group, and appropriateness. The vaccination offer has been extended to children between 6 months and 6 years of age, adults aged 60–64 years, and women in any trimester of pregnancy and postpartum. In parallel, from the 2018–2019 season, following the availability of new vaccines, the concept of vaccine appropriateness was introduced in the recommendations, i.e., the choice of the most appropriate vaccine for each subject to be vaccinated. The last circulars introduced indications of the type of vaccine that can be administered for each target category and the recommendation to use adjuvanted and high-dose vaccines for adults aged ≥ 65 years. The annual recommendations included in the Ministerial Circulars provided by the Italian Ministry of Health, resultantly, are increasingly precise, emphasizing the importance of the concept of appropriateness and outlining the path to precision vaccination.

In an ever-evolving field due to the growing availability of new types of vaccines and evidence of their immunogenicity, efficacy, effectiveness, and safety, it is essential that annual influenza vaccination recommendations evolve to allow the administration of the most appropriate vaccine to each target group. Only precision vaccination can bring maximum value to this preventive intervention, both in terms of clinical efficacy and societal benefits. This concept should continue to be considered as a basis for the development of future recommendations, as it addresses a critical public health issue related to vaccination.

## Figures and Tables

**Table 1:** Categories to whom seasonal influenza vaccination is recommended and actively and freely offered in Italy (without a priority order) [12].

	Target Population of Influenza Vaccination Program
**Subjects at high risk of complications or hospitalizations related to influenza:**	- Subjects aged 60 years or older - Women who are in any trimester of pregnancy at the beginning of the epidemic season and during the postpartum period - People from 7 years to 60 years of age affected by the following pathologies: *(a) Chronic diseases affecting the respiratory system (including severe asthma, bronchopulmonary dysplasia, cystic fibrosis, and chronic obstructive pulmonary disease—COPD);* *(b) Cardiovascular diseases, including congenital and acquired heart diseases;* *(c) Diabetes mellitus and other metabolic diseases (including obese individuals with a body mass index (BMI) >30);* *(d) Chronic kidney/adrenal failure;* *(e) Hematopoietic organ diseases and hemoglobinopathies;* *(f) Tumors and individuals undergoing chemotherapy;* *(g) Congenital or acquired diseases resulting in deficient antibody production, drug-induced immunosuppression, or HIV;* *(h) Chronic inflammatory diseases and intestinal malabsorption syndromes;* *(i) Pathologies for which major surgical interventions are planned;* *(j) Pathologies associated with an increased risk of the aspiration of respiratory secretions* (e.g., *neuromuscular diseases*); *(k) Chronic liver diseases.* - Children aged 6 months–6 years - Children and adolescents on long-term treatment with acetylsalicylic acid, at risk of Reye’s syndrome in case of influenza infection - Individuals of any age hospitalized in long-stay facilities - Family members and contacts (adults and children) of individuals at high risk of complications (regardless of whether the at-risk individual has been vaccinated or not)
**Subjects employed in public services of primary collective interest and categories of workers:**	- Doctors and healthcare/social care personnel in facilities - Police forces - Firefighters - Other socially useful categories that could benefit from vaccination, for reasons linked to the performance of their work activities
**Personnel who, for work-related reasons, come into contact with animals that could serve as a source of infection from non-human influenza viruses:**	- Farmers - Livestock workers - Livestock transporters - Slaughterers and vaccinators - Public and freelance veterinarians
**Other categories:**	- Blood donors

**Table 2:** Vaccines authorized by AIFA in Italy, indicated in Ministerial Circulars.

	Types of Vaccines Reported in the Annual Italian Circulars for the Prevention and Control of Influenza							
Influenza Season	Inactivated Split or Subunit Vaccine	Virosome-Adjuvanted Trivalent Inactivated Influenza Vaccine	Adjuvanted Inactivated Vaccine (MF59)	Trivalent Inactivated Intradermal Vaccine (Split)	Cell-Based Vaccine	Live-Attenuated Vaccine	High-Dose Quadrivalent Split Inactivated Vaccine	Recombinant Quadrivalent Vaccine
**2013–2014**	TIV	VATIIV	aTIV	IDTIIV				
**2014–2015**	TIV o QIV		aTIV	IDTIIV				
**2015–2016**	TIV o QIV		aTIV	IDTIIV	TIVcc			
**2016–2017**	TIV o QIV		aTIV	IDTIIV				
**2017–2018**	TIV o QIV		aTIV	IDTIIV				
**2018–2019**	TIV o QIV		aTIV					
**2019–2020**	TIV o QIV		aTIV		QIVcc			
**2020–2021**	TIV o QIV		aTIV		QIVcc		QIVhd	
**2021–2022**	QIV		aQIV		QIVcc	LAIV (quadrivalent)	QIVhd	rQIV
**2022–2023**	QIV		aQIV		QIVcc	LAIV (quadrivalent)	QIVhd	rQIV
**2023–2024**	QIV		aQIV		QIVcc	LAIV (quadrivalent)	QIVhd	rQIV
**2024–2025**	QIV		aQIV		QIQcc	LAIV (trivalent)	QIVhd	rQIV

Legend: TIV: trivalent inactivated vaccine; QIV: quadrivalent inactivated vaccine; VATIIV: virosome-adjuvanted trivalent inactivated influenza vaccine; aTIV: adjuvanted inactivated trivalent vaccine; aQIV: adjuvanted inactivated quadrivalent vaccine; IDTIIV: trivalent inactivated intradermal vaccine; TIVcc: cell-based trivalent vaccine; QIVcc: cell-based quadrivalent vaccine; LAIV: live-attenuated vaccine; QIVhd: high-dose quadrivalent split inactivated vaccine; rQIV: recombinant quadrivalent vaccine.

**Table 3:** Types of influenza vaccines administrable by age group, according to the SPC, reported in the annual Italian circulars for the prevention and control of influenza.

Influenza Season	Age Groups				
	6 Months– 9 Years Old	10–17 Years Old	18–59 Years Old	60–64 Years Old	≥65 Years Old
**2013–2014**	TIV VATIIV	TIV VATIIV	TIV VATIIV IDTIIV	TIV VATIIV IDTIIV	TIV VATIIV IDTIIV aTIV
**2014–2015**	TIV or QIV	TIV or QIV	TIV or QIV IDTIIV	TIV or QIV IDTIIV	TIV or QIV aTIV
**2015–2016**	TIV or QIV	TIV or QIV	TIV or QIV TIVcc	TIV or QIV IDTIIV TIVcc	TIV or QIV IDTIIV TIVcc aTIV
**2016–2017**	TIV or QIV	TIV or QIV	TIV or QIV	TIV or QIV IDTIIV	TIV or QIV IDTIIV aTIV
**2017–2018**	TIV or QIV	TIV or QIV	TIV or QIV	TIV or QIV IDTIIV	TIV or QIV IDTIIV aTIV
**2018–2019**	TIV or QIV	TIV or QIV	TIV or QIV	TIV or QIV	TIV or QIV aTIV
**2019–2020**	TIV or QIV	TIV or QIV QIVcc	TIV or QIV QIVcc	TIV or QIV QIVcc	TIV or QIV QIVcc aTIV
**2020–2021**	TIV or QIV	TIV or QIV QIVcc	TIV or QIV QIVcc	TIV or QIV QIVcc	TIV or QIV QIVcc aTIV QIVhd
**2021–2022**	QIV QIVcc (≥2 years) LAIV (≥2 years)	QIV QIVcc LAIV	QIV QIVcc rQIV	QIV QIVcc rQIV	QIV QIVcc rQIV QIVhd aQIV
**2022–2023**	QIV QIVcc (≥2 years) LAIV (≥2 years)	QIV QIVcc LAIV	QIV QIVcc rQIV QIVhd	QIV QIVcc rQIV QIVhd	QIV QIVcc rQIV QIVhd aQIV
**2023–2024**	QIV QIVcc (≥2 years) LAIV (≥2 years)	QIV QIVcc LAIV	QIV QIVcc rQIV	QIV QIVcc rQIV QIVhd	QIV QIVcc rQIV QIVhd aQIV
**2024–2025**	QIV QIVcc (≥2 years) LAIV (≥2 years)	QIV QIVcc LAIV	QIV QIVcc rQIV aQIV (≥50 years)	QIV QIVcc rQIV QIVhd aQIV	QIV QIVcc rQIV QIVhd aQIV

Legend: TIV: trivalent inactivated vaccine; QIV: quadrivalent inactivated vaccine; VATIIV: virosome-adjuvanted trivalent inactivated influenza vaccine; aTIV: adjuvanted inactivated trivalent vaccine; aQIV: adjuvanted inactivated quadrivalent vaccine; IDTIIV: trivalent inactivated intradermal vaccine; TIVcc: cell-based trivalent vaccine; QIVcc: cell-based quadrivalent vaccine; LAIV: live-attenuated vaccine; QIVhd: high-dose quadrivalent split inactivated vaccine; rQIV: recombinant quadrivalent vaccine.

**Table 4:** Vaccines administrable and recommended for each target population according to the Ministerial Circular for the 2024–2025 season [12].

Target Population	Influenza Vaccines					
	QIV	aQIV	rQIV	QIVhd	LAIV	QIVcc
Subjects aged 65 years or older	A	R	A	R		A
People in the 60–64 years age group	A	A	A	A		A
Subjects aged between 50 years and 59 years who are included in the categories listed in Table 1	A	A	A			A
Subjects aged between 18 years and 49 years who are included in the categories listed in Table 1	A		A			A
Children aged between 7 and 17 years who are included in the categories listed in Table 1	A				A	A
Children in the 2–6 years age group	A				A	A
Children in the 6 months–2 years age group	A					
Women who, at the beginning of the epidemic season, are in any trimester of pregnancy or the “postpartum” period	A		A			A

A: administrable according to SPC; R: recommended product to be administered.

**Table 5:** Summary of references and major results regarding the evidence for the preferential use of QIVhd and aQIV.

Reference	Authors	Type of Study	Results
[50]	Lee JKH et al. (2018)	Systematic review	High-dose inactivated trivalent influenza vaccine is more effective than standard-dose trivalent influenza vaccine at reducing the clinical outcomes associated with influenza infection in adults ≥ 65.
[51]	Lee JKH et al. (2023)	Systematic review	High-dose inactivated trivalent influenza vaccine is more effective than standard-dose trivalent influenza vaccine at reducing influenza and associated serious outcomes in people aged ≥ 65 years, irrespective of age or characteristics of the influenza season.
[52]	DiazGranados CA et al. (2014)	Phase IIIb-IV, multicenter, randomized, double-blind, active-controlled trial	Assessments of relative efficacy, effectiveness, safety, and immunogenicity were performed during the 2011–2012 and the 2012–2013 northern hemisphere influenza seasons. Among persons 65 years of age or older, high-dose inactivated trivalent influenza vaccine induced significantly higher antibody responses and provided better protection against laboratory-confirmed influenza illness than did standard-dose trivalent influenza vaccine.
[53]	Balasubramani GK et al. (2020)	Test-negative case–control study	US Flu Vaccine Effectiveness Network data from the 2015–2016 through 2018–2019 seasons were analyzed to determine relative vaccine effectiveness between high-dose inactivated trivalent influenza vaccine and standard-dose trivalent influenza vaccine among outpatients ≥ 65 years old presenting with acute respiratory illness. High-dose vaccine offered more protection against A/H3N2 and borderline significant protection against all influenza A infections requiring outpatient care during the 2015–2018 influenza.
[54]	Doyle JD et al. (2021)	Observational study	Hospitalized patients with acute respiratory illness were enrolled in an observational vaccine effectiveness study during the 2015–2016 and 2016–2017 influenza seasons. Enrolled patients were tested for influenza, and receipt of influenza vaccine by type was recorded. Effectiveness of standard-dose vaccine and high-dose vaccine was estimated using a test-negative design. High-dose vaccine offered greater effectiveness.
[58]	Li APY et al. (2021)	Randomized controlled trial	The cellular and antibody responses of standard-dose vaccines versus enhanced vaccines, MF59-adjuvanted, high-dose, and recombinant vaccines were compared in adults ≥ 65. Enhanced (high-dose and adjuvanted) influenza vaccines increase the polyfunctional CD4+ and CD8+ T-cell response.

## Data Availability

Information on the revised Ministerial Circulars is available on the official website of the Italian Ministry of Health: https://www.salute.gov.it/portale/home.html (accessed on 24 March 2025).

## References

[1] World Health Organization (WHO) Influenza (Seasonal). https://www.who.int/news-room/fact-sheets/detail/influenza-(seasonal).

[2] World Health Organization (WHO) Global Influenza Strategy 2019–2030. https://www.who.int/publications/i/item/9789241515320.

[3] European Centre for Disease Prevention and Control Factsheet About Seasonal Influenza. https://www.ecdc.europa.eu/en/seasonal-influenza/facts/factsheet.

[4] Epicentro Influenza. General Information. https://www.epicentro.iss.it/influenza/influenza.

[5] Hayward A.C., Fragaszy E.B., Bermingham A., Wang L., Copas A., Edmunds W.J., Ferguson N., Goonetilleke N., Harvey G., Kovar J. (2014). Comparative community burden and severity of seasonal and pandemic influenza: Results of the Flu Watch cohort study. Lancet Respir. Med..

[6] People at Increased Risk for Flu Complications Centers for Disease Control and Prevention. https://www.cdc.gov/flu/highrisk/index.htm.

[7] Boccalini S., Bechini A. (2024). Assessment of Epidemiological Trend of Influenza-Like Illness in Italy from 2010/2011 to 2023/2024 Season: Key Points to Optimize Future Vaccination Strategies against Influenza. Vaccines.

[8] Hall E. (2021). Influenza. The Pink Book.

[9] Boccalini S., Bechini A., Innocenti M., Sartor G., Manzi F., Bonanni P., Panatto D., Lai P.L., Zangrillo F., Rizzitelli E. (2018). La vaccinazione universale dei bambini contro l’influenza con il vaccino Vaxigrip Tetra^®^ in Italia: Risultati di una valutazione di Health Technology Assessment (HTA) [The universal influenza vaccination in children with Vaxigrip Tetra^®^ in Italy: An evaluation of Health Technology Assessment]. J. Prev. Med. Hyg..

[10] SItI, SIP, FIMP, FIMG, SIMG Vaccine Calendar for Life, 2025. V Edition. [Italian] Calendario Vaccinale per la vita. https://www.simg.it/wp-content/uploads/2024/12/calendario-per-la-vita-2025.pdf.

[11] Bonanni P., Boccalini S., Zanobini P., Dakka N., Lorini C., Santomauro F., Bechini A. (2018). The appropriateness of the use of influenza vaccines: Recommendations from the latest seasons in Italy. Hum. Vaccines Immunother..

[12] Ministry of Health Influenza Prevention and Control: Recommendations for the Season 2024–2025. https://www.trovanorme.salute.gov.it/norme/renderNormsanPdf?anno=2024&codLeg=100738&parte=1%20&serie=null.

[13] Ministry of Health. Influenza. https://www.salute.gov.it/new/it/tema/influenza/.

[14] Ministry of Health Influenza Prevention and Control: Recommendations for the Season 2013–2014. https://www.trovanorme.salute.gov.it/norme/renderNormsanPdf?anno=0&codLeg=46769&parte=1%20&serie=.

[15] Ministry of Health Influenza Prevention and Control: Recommendations for the Season 2014–2015. https://www.trovanorme.salute.gov.it/norme/renderNormsanPdf?anno=0&codLeg=49871&parte=1%20&serie=.

[16] Ministry of Health Influenza prevention and Control: Recommendations for the Season 2015–2016. https://www.trovanorme.salute.gov.it/norme/renderNormsanPdf?anno=0&codLeg=52703&parte=1%20&serie=.

[17] Ministry of Health Influenza prevention and Control: Recommendations for the Season 2016–2017. https://www.trovanorme.salute.gov.it/norme/renderNormsanPdf?anno=2016&codLeg=55586&parte=1%20&serie=null.

[18] Ministry of Health Influenza Prevention and Control: Recommendations for the Season 2017–2018. https://www.trovanorme.salute.gov.it/norme/renderNormsanPdf;jsessionid=JCEbZYZ6VGHBhyATrnouzg__.sgc4-prd-sal?anno=2017&codLeg=60180&parte=1%20&serie=null.

[19] Ministry of Health Influenza Prevention and Control: Recommendations for the Season 2018–2019. https://www.trovanorme.salute.gov.it/norme/renderNormsanPdf?anno=2018&codLeg=64381&parte=1%20&serie=null.

[20] Ministry of Health Influenza Prevention and Control: Recommendations for the Season 2019–2020. https://www.trovanorme.salute.gov.it/norme/renderNormsanPdf?anno=2019&codLeg=70621&parte=1%20&serie=null.

[21] Ministry of Health Influenza Prevention and Control: Recommendations for the Season 2020–2021. https://www.trovanorme.salute.gov.it/norme/renderNormsanPdf?anno=2020&codLeg=74451&parte=1%20&serie=null.

[22] Ministry of Health Influenza Prevention and Control: Recommendations for the Season 2021–2022. https://www.trovanorme.salute.gov.it/norme/renderNormsanPdf?anno=2021&codLeg=79647&parte=1%20&serie=null.

[23] Ministry of Health Influenza Prevention and Control: Recommendations for the Season 2022–2023. https://www.trovanorme.salute.gov.it/norme/renderNormsanPdf?anno=2022&codLeg=87997&parte=1%20&serie=null.

[24] Ministry of Health Influenza Prevention and Control: Recommendations for the Season 2023–2024. https://www.trovanorme.salute.gov.it/norme/renderNormsanPdf?anno=2023&codLeg=93294&parte=1%20&serie=null.

[25] Ministry of Health Recommended Vaccinations for Women of Reproductive Age And pregnancy. Update November 2019. https://www.trovanorme.salute.gov.it/norme/renderNormsanPdf?anno=2019&codLeg=71540&parte=1%20&serie=null.

[26] WHO Recommended Composition of Influenza Virus Vaccines for Use in the 2024–2025 Northern Hemisphere Influenza Season. 23 February 2024. https://www.who.int/publications/m/item/recommended-composition-of-influenza-virus-vaccines-for-use-in-the-2024-2025-northern-hemisphere-influenza-season.

[27] Dipartimento della Programmazione e dell’Ordinamento del Servizio Sanitario Nazionale; Direzione Generale della Programmazione Sanitaria Ufficio III ex D.G.PROGS. Manuale di formazione per il governo clinico: Appropriatezza; 2012. https://www.salute.gov.it/imgs/C_17_pubblicazioni_1826_allegato.pdf.

[28] WHO Regional Office for Europe Appropriateness in Health Care Services. Report on a WHO Workshop, Koblenz, Germany 23–25 March 2000. https://iris.who.int/handle/10665/108350.

[29] Kheiraoui F., Cadeddu C., Quaranta G., Poscia A., Raponi M., de Waure C., Boccalini S., Pellegrino E., Bellini I., Pieri L. (2015). Health Technology Assessment del vaccino antinfluenzale quadrivalente FLU-QIV (Fluarix Tetra^®^). Ital. J. Public Health.

[30] Di Pietro M.L., Poscia A., Specchia M.L., de Waure C., Zace D., Gasparini R., Amicizia D., Lai P.L., Panatto D., Arata L. (2017). Valutazione di Health Technology Assessment (HTA) del vaccino antinfluenzale adiuvato nella popolazione anziana italiana. Ital. J. Public Health.

[31] Calabrò G.E., Boccalini S., Del Riccio M., Ninci A., Manzi F., Bechini A., Bonanni P., Panatto D., Lai P.L., Amicizia D. (2019). Valutazione di Health Technology Assessment (HTA) del vaccino antinfluenzale quadrivalente da coltura cellulare: Flucelvax Tetra. Ital. J. Public Health.

[32] Calabrò G.E., Boccalini S., Bonanni P., Bechini A., Panatto D., Lai P.L., Amicizia D., Rizzo C., Ajelli M., Trentini F. (2021). Valutazione di Health Technology Assessment (HTA) del vaccino antinfluenzale quadrivalente adiuvato: Fluad Tetra. Ital. J. Public Health.

[33] Cicchetti A., Rumi F., Basile M., Orsini F., Gualano M.R., Bert F., Orsi A., Refolo P., Sacchini D., Casini M. (2021). Report HTA del vaccino quadrivalente ad alto dosaggio (QIV-HD) EFLUELDA^®^ per la prevenzione dell’influenza stagionale e delle sue complicanze nella popolazione over 65. Ital. J. Public Health.

[34] Boccalini S., Pariani E., Calabrò G.E., De Waure C., Panatto D., Amicizia D., Lai P.L., Rizzo C., Amodio E., Vitale F. (2021). Health Technology Assessment (HTA) dell’introduzione della vaccinazione antinfluenzale per la popolazione giovanile italiana con il vaccino Fluenz Tetra^®^. J. Prev. Med. Hyg..

[35] Calabrò G.E., Boccalini S., Bechini A., Panatto D., Domnich A., Lai P.L., Amicizia D., Rizzo C., Pugliese A., Di Pietro M.L. (2022). L’Health Technology Assessment come strumento value based per la valutazione delle tecnologie sanitarie. Reassessment del vaccino antinfluenzale quadrivalente da coltura cellulare: Flucelvax Tetra® 2.0. J. Prev. Med. Hyg..

[36] Iob A., Brianti G., Zamparo E., Gallo T. (2005). Evidence of increased clinical protection of an MF-59-adjuvant influenza vaccine compared to a non adjuvant vaccine among elderly residents of long term care facilities in Italy. Epidemiol. Infect..

[37] Ansaldi F., Zancolli M., Durando P., Montomoli E., Sticchi L., Del Giudice G., Icardi G. (2010). Antibody response against heterogeneous circulating influenza virus strains elicited by MF59- and non-adjuvanted vaccines during seasons with good or partial matching between vaccine strain and clinical isolates. Vaccine.

[38] Mannino S., Villa M., Apolone G., Weiss N.S., Groth N., Aquino I., Boldori L., Caramaschi F., Gattinoni A., Malchiodi G. (2012). Effectiveness of adjuvanted influenza vaccination in elderly subjects in Northern Italy. Am. J. Epidemiol..

[39] Camilloni B., Neri M., Lepri E., Iorio A.M. (2009). Cross-reactive antibodies in middle-aged and elderly volunteers after MF59-adjuvanted subunit trivalent influenza vaccine against B viruses of the B/Victoria or B/Yamagata lineages. Vaccine.

[40] Van Buynder P.G., Konrad S., Van Buynder J.L., Brodkin E., Krajden M., Ramler G., Bigham M. (2013). The comparative effectiveness of adjuvanted and unadjuvanted trivalent inactivated influenza vaccine (TIV) in the elderly. Vaccine.

[41] Mira-Iglesias A., López-Labrador F.X., Baselga-Moreno V., Tortajada-Girbés M., Mollar-Maseres J., Carballido-Fernández M., Schwarz-Chavarri G., Puig-Barberà J., Díez-Domingo J. (2019). Influenza vaccine effectiveness against laboratory-confirmed influenza in hospitalised adults aged 60 years or older, Valencia Region, Spain, 2017/18 influenza season. Eurosurveillance.

[42] Pebody R., Whitaker H., Zhao H., Andrews N., Ellis J., Donati M., Zambon M. (2020). Protection provided by influenza vaccine against influenza-related hospitalisation in ≥65 year olds: Early experience of introduction of a newly licensed adjuvanted vaccine in England in 2018/19. Vaccine.

[43] Pebody R.G., Whitaker H., Ellis J., Andrews N., Marques D.F.P., Cottrell S., Reynolds A.J., Gunson R., Thompson C., Galiano M. (2020). End of season influenza vaccine effectiveness in primary care in adults and children in the United Kingdom in 2018/19. Vaccine.

[44] Rondy M., Larrauri A., Casado I., Alfonsi V., Pitigoi D., Launay O., Syrjänen R.K., Gefenaite G., Machado A., Vučina V.V. (2017). 2015/16 seasonal vaccine effectiveness against hospitalisation with influenza a(H1N1)pdm09 and B among elderly people in Europe: Results from the I-MOVE+ project. Eurosurveillance.

[45] Hartvickson R., Cruz M., Ervin J., Brandon D., Forleo-Neto E., Dagnew A.F., Chandra R., Lindert K., Mateen A.A. (2015). Non-inferiority of mammalian cell-derived quadrivalent subunit influenza virus vaccines compared to trivalent subunit influenza virus vaccines in healthy children: A phase III randomized, multicenter, double-blind clinical trial. Int. J. Infect. Dis..

[46] Bart S., Cannon K., Herrington D., Mills R., Forleo-Neto E., Lindert K., Abdul Mateen A. (2016). Immunogenicity and safety of a cell culture- based quadrivalent influenza vaccine in adults: A Phase III, double-blind, multicenter, randomized, non-inferiority study. Hum. Vaccines Immunother..

[47] Bruxvoort K.J., Luo Y., Ackerson B., Tanenbaum H.C., Sy L.S., Gandhi A., Tseng H.F. (2019). Comparison of vaccine effectiveness against influenza hospitalization of cell-based and egg-based influenza vaccines, 2017–2018. Vaccine.

[48] Klein N.P., Fireman B., Goddard K., Zerbo O., Asher J., Zhou J., King J., Lewis N. (2020). Vaccine effectiveness of cell-culture relative to egg-based inactivated influenza vaccine during the 2017−18 influenza season. PLoS ONE.

[49] Martin E.T., Cheng C., Petrie J.G., Alyanak E., Gaglani M., Middleton D.B., Ghamande S., Silveira F.P., Murthy K., Zimmerman R.K. (2021). Low Influenza Vaccine Effectiveness Against A(H3N2)-Associated Hospitalizations in 2016−2017 and 2017−2018 of the Hospitalized Adult Influenza Vaccine Effectiveness Network (HAIVEN). J. Infect. Dis..

[50] Lee J.K.H., Lam G.K.L., Shin T., Kim J., Krishnan A., Greenberg D.P., Chit A. (2018). Efficacy and effectiveness of high-dose versus standard-dose influenza vaccination for older adults: A systematic review and meta-analysis. Expert Rev. Vaccines.

[51] Lee J.K.H., Lam G.K.L., Yin J.K., Loiacono M.M., Samson S.I. (2023). High-dose influenza vaccine in older adults by age and seasonal characteristics: Systematic review and meta-analysis update. Vaccine X.

[52] DiazGranados C.A., Dunning A.J., Kimmel M., Kirby D., Treanor J., Collins A., Pollak R., Christoff J., Earl J., Landolfi V. (2014). Efficacy of high-dose versus standard-dose influenza vaccine in older adults. N. Engl. J. Med..

[53] Balasubramani G.K., Choi W.S., Nowalk M.P., Zimmerman R.K., Monto A.S., Martin E.T., Belongia E.A., McLean H.Q., Gaglani M., Murthy K. (2020). Relative effectiveness of high dose versus standard dose influenza vaccines in older adult outpatients over four seasons, 2015−16 to 2018−19. Vaccine.

[54] Doyle J.D., Beacham L., Martin E.T., Talbot H.K., Monto A., Gaglani M., Middleton D.B., Silveira F.P., Zimmerman R.K., Alyanak E. (2021). Relative and Absolute Effectiveness of High-Dose and Standard-Dose Influenza Vaccine Against Influenza-Related Hospitalization Among Older Adults-United States, 2015–2017. Clin. Infect. Dis. Off. Publ. Infect. Dis. Soc. Am..

[55] Dunkle L.M., Izikson R., Patriarca P., Goldenthal K.L., Muse D., Callahan J., Cox M.M. (2017). Efficacy of recombinant influenza vaccine in adults 50 years of age or older. N. Engl. J. Med..

[56] Zimmerman R.K., Dauer K., Clarke L., Nowalk M.P., Raviotta J.M., Balasubramani G.K. (2023). Vaccine effectiveness of recombinant and standard dose influenza vaccines against outpatient illness during 2018-2019 and 2019-2020 calculated using a retrospective test-negative design. Hum. Vaccines Immunother..

[57] Dunkle L.M., Izikson R., Patriarca P.A., Goldenthal K.L., Muse D., Cox M.M.J. (2017). Randomized comparison of immunogenicity and safety of quadrivalent recombinant versus inactivated influenza vaccine in healthy adults 18-49 years of age. J. Infect. Dis..

[58] Li A.P.Y., Cohen C.A., Leung N.H.L., Fang V.J., Gangappa S., Sambhara S., Levine M.Z., Iuliano A.D., Perera R.A.P.M., Ip D.K.M. (2021). Immunogenicity of standard, high-dose, MF59-adjuvanted, and recombinant-HA seasonal influenza vaccination in older adults. NPJ Vaccines.

[59] WHO Vaccine Against Influenza: WHO Position Paper–May 2022. https://iris.who.int/bitstream/handle/10665/354264/WER9719-eng-fre.pdf?sequence=1.

[60] Recommendations by the Standing Committee on Vaccination (STIKO) at the Robert Koch Institute–2023. https://www.rki.de/EN/Topics/Infectious-diseases/Immunisation/STIKO/STIKO-recommendations/stiko-recommendations-node.html.

[61] Australian Government Department of Health and Aged Care. Statement on the Administration of Seasonal Influenza Vaccines in 2024. https://www.health.gov.au/sites/default/files/2024-02/atagi-statement-on-the-administration-of-seasonal-influenza-vaccines-in-2024.pdf.

[62] National Immunisation Programme 2024, UK. https://www.gov.uk/government/publications/national-flu-immunisation-programme-plan-2024-to-2025/national-flu-immunisation-programme-2024-to-2025-letter.

[63] Joint Committee on Vaccination and Immunisation Minute of the Meeting on 07 June 2023. https://app.box.com/s/iddfb4ppwkmtjusir2tc/file/1262409204637.

[64] Izurieta H.S., Lu M., Kelman J., Lu Y., Lindaas A., Loc J., Pratt D., Wei Y., Chillarige Y., Wernecke M. (2021). Comparative Effectiveness of Influenza Vaccines Among US Medicare Beneficiaries Ages 65 Years and Older During the 2019–2020 Season. Clin. Infect. Dis..

[65] Grohskopf L.A., Blanton L.H., Ferdinands J.M., Chung J.R., Broder K.R., Talbot H.K. (2023). Prevention and Control of Seasonal Influenza with Vaccines: Recommendations of the Advisory Committee on Immunization Practices—United States, 2023–2024 Influenza Season. MMWR Recomm. Rep..

[66] An Advisory Committee Statement (ACS) National Advisory Committee on Immunization (NACI) Statement on Seasonal Influenza Vaccine for 2023–2024. https://www.canada.ca/en/public-health/services/publications/vaccines-immunization/national-advisory-committee-immunization-statement-seasonal-influenza-vaccine-2023-2024.html.

[67] Ministry of Health Piano Nazionale Prevenzione Vaccinale 2023–2025. https://www.trovanorme.salute.gov.it/norme/dettaglioAtto.spring?id=95963&page=newsett.

[68] Ministry of Health Influenza Vaccination Coverage Data. https://www.salute.gov.it/portale/influenza/dettaglioContenutiInfluenza.jsp?lingua=italiano&id=679&area=influenza&menu=vuoto.

[69] Boccalini S., Tacconi F.M., Lai P.L., Bechini A., Bonanni P., Panatto D. (2019). Appropriateness and preferential use of different seasonal influenza vaccines: A pilot study on the opinion of vaccinating physicians in Italy. Vaccine.

[70] Bellino S., Piovesan C., Bella A., Rizzo C., Pezzotti P., Ramigni M. (2020). Determinants of vaccination uptake, and influenza vaccine effectiveness in preventing deaths and hospital admissions in the elderly population; Treviso, Italy, 2014/2015-2016/2017 seasons. Hum. Vaccines Immunother..

[71] Buja A., Grotto G., Taha M., Cocchio S., Baldo V. (2023). Use of Information and Communication Technology Strategies to Increase Vaccination Coverage in Older Adults: A Systematic Review. Vaccines.

[72] Stuurman A.L., Ciampini S., Vannacci A., Bella A., Rizzo C., Muñoz-Quiles C., Pandolfi E., Liyanage H., Haag M., Redlberger-Fritz M. (2021). Factors driving choices between types and brands of influenza vaccines in general practice in Austria, Italy, Spain and the UK. PLoS ONE.

[73] Ecarnot F., Maggi S., Michel J.P. (2020). Strategies to Improve Vaccine Uptake throughout Adulthood. Interdiscip. Top. Gerontol. Geriatr..

[74] Lanza T.E., Paladini A., Marziali E., Gianfredi V., Blandi L., Signorelli C., Odone A., Ricciardi W., Damiani G., Cadeddu C. (2023). Training needs assessment of European frontline health care workers on vaccinology and vaccine acceptance: A systematic review. Eur. J. Public Health.

[75] Capodici A., Montalti M., Soldà G., Salussolia A., La Fauci G., Di Valerio Z., Scognamiglio F., Fantini M.P., Odone A., Costantino C. (2023). Influenza vaccination landscape in Italy: A comprehensive study through the OBVIOUS project lens. Hum Vaccines Immunother..

[76] Holford D., Anderson E.C., Biswas A., Garrison A., Fisher H., Brosset E., Gould V.C., Verger P., Lewandowsky S. (2024). Healthcare professionals’ perceptions of challenges in vaccine communication and training needs: A qualitative study. BMC Prim. Care..

